# Two-dimensional analysis provides molecular insight into flower scent of *Lilium* ‘Siberia’

**DOI:** 10.1038/s41598-018-23588-9

**Published:** 2018-03-29

**Authors:** Shaochuan Shi, Guangyou Duan, Dandan Li, Jie Wu, Xintong Liu, Bo Hong, Mingfang Yi, Zhao Zhang

**Affiliations:** 1 Beijing Key Laboratory of Development and Quality Control of Ornamental Crops, Department of Ornamental Horticulture, China Agricultural University, Beijing, China; 2 Energy Plant Research Center, School of Life Sciences, Qilu Normal University, Jinan, China

## Abstract

Lily is a popular flower around the world not only because of its elegant appearance, but also due to its appealing scent. Little is known about the regulation of the volatile compound biosynthesis in lily flower scent. Here, we conducted an approach combining two-dimensional analysis and weighted gene co-expression network analysis (WGCNA) to explore candidate genes regulating flower scent production. In the approach, changes of flower volatile emissions and corresponding gene expression profiles at four flower developmental stages and four circadian times were both captured by GC-MS and RNA-seq methods. By overlapping differentially-expressed genes (DEGs) that responded to flower scent changes in flower development and circadian rhythm, 3,426 DEGs were initially identified to be candidates for flower scent production, of which 1,270 were predicted as transcriptional factors (TFs). The DEGs were further correlated to individual flower volatiles by WGCNA. Finally, 37, 41 and 90 genes were identified as candidate TFs likely regulating terpenoids, phenylpropanoids and fatty acid derivatives productions, respectively. Moreover, by WGCNA several genes related to auxin, gibberellins and ABC transporter were revealed to be responsible for flower scent production. Thus, this strategy provides an important foundation for future studies on the molecular mechanisms involved in floral scent production.

## Introduction

Flower scent attracts and guides pollinators to angiosperms to aid in fertilization^1^. In some cases, it also functions in plant defense^2^. Flower scent also brings people mental pleasure and provides essential oils and flavors used in the food and perfume industries^3^. Three classes of volatile compounds dominate flower scent: terpenoids, phenylpropanoids/benzenoids, and fatty-acid derivatives^4^. Terpenoids, including monoterpenoids, sesquiterpenoids, and diterpenes, compose the largest group of compounds responsible for flower scent^5^. Terpenoids are derived from dimethylallyl pyrophosphate (DMAPP) and isopentenyl pyrophosphate (IPP), which are condensed by prenyltransferases into geranyl pyrophosphate (GPP) and farnesyl pyrophosphate (FPP), two direct precursors of various terpenoid products^6^. Two divergent pathways are involved in biosynthesis of terpenoids: the methylerythritol phosphate (MEP) pathway operates in the plastids^7^ and is responsible for mono- and diterpene production^8^, whereas the mevalonic acid (MVA) pathway occurs in the cytosol, endoplasmic reticulum, and peroxisome^9,10^ and gives rise to volatile sesquiterpenes. Research has mostly focused on the genes involved in the final steps of terpenoid biosynthesis, and more than 100 terpene synthases (TPSs) genes have been identified in a variety of plant species^11^.

Phenylpropanoids/benzenoids represent the second largest class of flower scent compounds^12^, and are exclusively derived from the aromatic amino acid L-phenylalanine (L-Phe). In one branch of the pathway, phenylpropanoid-related compounds originate directly from L-Phe. L-Phe is converted into phenylacetaldehyde (PAA) by aromatic amino acid decarboxylase (AADC) or phenylacetaldehyde synthase (PAAS^13–15^) and then converted to 2-phenylethanol (2-PE) by phenyl acetaldehyde reductase (PAR^16,17^). Biosynthesis of benzenoids depends on another branch of the phenylpropanoid pathway: the cinnamic acid pathway^18^. Genes involved in biosynthesis of 3,5-dimethoxy toluene (DMT) and 1,3,5-trimethoxy benzene (TMB) have been isolated from roses, such as *RcPOMT* for phloroglucinol O-methyltransferase (POMT^19^) and *RcOOMT1* and *RcOOMT2* for two orcinol O-methyltransferases^20,21^. Genes for (iso)eugenol and methyl(iso)eugenol have also been identified in *Rosa chinensis*^22^, *Petunia hybrida*^23^, and *Clarkia breweri*^24^.

Fatty acid derivatives constitute the third class of flower volatile compounds, and are initiated by a stereo-specific oxygenation of octadecanoid precursors and subsequently produced by the lipoxygenase (LOX) pathway. The formation of 9- and 13-hydroperoxy intermediates results in branches to hydroperoxide lyase and allene oxide synthase (AOS)^25,26^. In the AOS branch, the 13-hydroperoxy intermediate is ultimately converted to jasmonic acid (JA), while in the hydroperoxide lyase branch both 9- and 13-hydroperoxy intermediates are converted into volatile C6 and C9 aldehydes and alcohols, which are commonly referred to as green leaf volatiles.

Although an increasing number of genes for flower volatile biosynthesis have been cloned, the complete regulatory mechanism is still unknown. Several transcription factors (TFs) has been identified, including four R2R3-type MYB TFs, *ODO1*^27^, *EOBI*^28,29^, *EOBII*^29–31^, and *PH4*^32^ in petunia, and two repressor TFs, *PhMYB4*^33^ in petunia and *MYB3* in arabidopsis^34^, which all regulate gene expression of phenylpropanoid/benzenoid production. By contrast, only two TFs were identified for transcriptional regulation of the terpenoids in arabidopsis and citrus^35,36^, while no one was identified for fatty acid derivatives.

Lily is the fourth most popular cut flower worldwide, prized for both its appearance and its fragrance. Terpenoids and benzenoids, such as linalool, (E)-β-ocimene, myrcene, 1,8-cineole, isoeugenol, benzyl alcohol, ethyl benzoate, and methyl benzoate, are abundant in lily flower aromas^37–39^. However, little is known about the molecular mechanism underlying the production of these flower volatiles. To date, only two monoterpene synthases^40,41^ and one benzoic acid/salicylic acid carboxyl methyltransferase^42^ genes have been isolated from lily. In addition, recent studies have shown that scent emissions of lily is in concert with floral development stages^41^, and exhibited rhythmic patterns^37^.

In *Lilium*, RNA-seq has been used to analyze carbohydrate metabolism^43^, generative cell formation^44,45^, flavonoid biosynthesis^46^, cold response, and signal pathways^47,48^. However, an obstacle remains: it is intractable to identify the exact candidate genes because thousands of differentially-expressed genes (DEGs) are usually obtained.

In the present study, we used a strategy combining two-dimensional (2-D) with a weighted gene co-expression network analysis (WGCNA) method to identify critical genes involved in flower scent production of one popular lily cultivar *Lilium* ‘Siberia’. With this strategy, we captured flower scent and gene expression changes in two dimensions, including four flowering stages as well as four times throughout the day during full bloom. The DEGs from the two processes were overlapped to eliminate the irrelevant ones and then further screened and verified by WGCNA analysis. This combined strategy allowed us to decrease the DEG number and identify candidate genes (TFs) responsible for flower scent production efficiently.

## Materials and Methods

### Plant material

**Figure 1:**
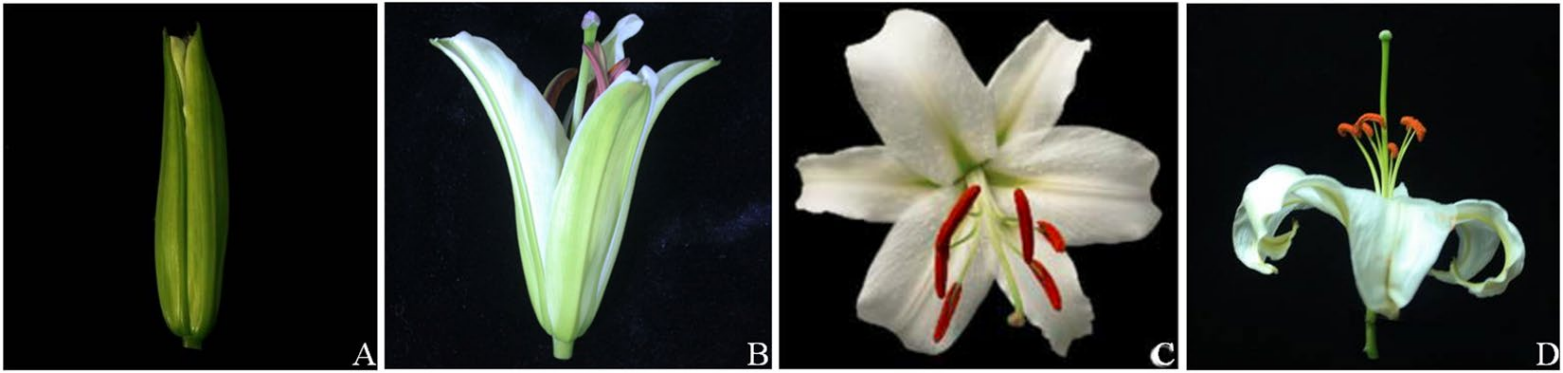
Flower developmental stages in *Lilium* ‘Siberia’. (**A**) Early-flowering (EF): tepals splitting slightly, the flower color changing from green to white; **(B)** Semi-flowering (SF): tepals semi-opening, the flower color becoming white; **(C)** Full-flowering (FF): tepals full-opening, white flower color; **(D)** Late-flowering (LF): tepals wilting, the flower color becoming bleak.

Fresh flowers of *Lilium* ‘Siberia’ were obtained from a local farm in Haidian district, Beijing. Flower development was divided into four stages: early flowering (EF), semi flowering (SF), full flowering (FF) and late flowering (LF; Fig. 1A–D). For the analyses related to the developmental stages, all flower samples were collected from greenhouse at 16:00 (temperature: 18 °C). For the analyses related to the circadian rhythm of flower scent, flowers were harvested from greenhouse and placed immediately in water. The flowers were then delivered to the laboratory and kept in an illuminating incubator for one day (temperature: 26 °C; photoperiod: 12 h, from 08:00 to 20:00)^37,39^. Flowers were then sampled four times in one day: 04:00, 10:00, 16:00, and 22:00. For every sample, half of the flower material was used for GC-MS analysis and the other half was immediately frozen in liquid nitrogen and stored at −80 °C for RNA-seq analysis. Three replicates were prepared for every sample, respectively.

### Floral scent collection and GC-MS analysis

For every sample, 3 g of sepals were collected and inserted into a 100-mL sample vial with ethyl caprate (10 μL, 0.865 μg·μL^−1^; Sigma Ltd. Co., USA) as an internal standard. A headspace solid-phase microextraction (SPME) manual headspace sampler with a 100 μm polydimethylsiloxane (PDMS) fiber (Supelco, USA) was used to extract and concentrate the headspace floral volatiles in the vial. After extraction at 30 °C for 40 min, GC-MS was conducted (Trace DSQ-GC-MS; Thermo Corporation, USA). The GC was fitted with a DB-5MS fused-silica capillary column (30 m × 0.25 mm × 0.25 mm film) with helium (99.99%) as the carrier gas at a flow rate of 1.00 mL·min^−1^. The column temperature was programmed as follows: the injection port was set to 250 °C; the GC oven was maintained at 50 °C for 1 min, increased to 200 °C at a rate of 5 °C·min^−1^ then increased to 230 °C at 8 °C·min^−1^, and finally maintained at 230 °C for 8 min. Compounds were identified by matching the mass spectra with the NIST 11 library (National Institute of Standards and Technology, Gaithersburg, MD, USA) and retention index. Quantitative analysis was conducted with peak areas of volatile compounds and the internal standard^49^. The mass fraction was calculated using the formula given below: compound emission rate (μg·g^−1^·h^−1^) = {peak area of compound/peak area of internal standard × concentration of internal standard (μg·μL^−1^) × volume of internal standard}/sample mass (g)/extraction time (h).

### RNA extraction, library preparation, and sequencing

Total RNA was extracted from]flowers using an RNAprep pure kit (Tiangen Biotech Co., Ltd., Beijing, China) following the manufacturer’s protocol. RNA degradation and contamination was detected by 1% agarose gel electrophoresis. A spectrophotometer (NanoPhotometer; Implen, CA, USA) was used to check RNA purity, and a Qubit RNA assay kit and a Qubit 2.0 fluorometer (Life Technologies, Carlsbad, CA, USA) were used to confirm its concentration. RNA integrity was measured using an RNA 6000 Nano Assay kit on a Bioanalyzer 2100 system (Agilent, Carlsbad, CA, USA).

Sequencing libraries were generated from total RNA using a NEBNext Ultra Directional RNA library prep kit for Illumina following the manufacturer’s instructions (NEB, Ipswich, MA, USA). The library preparations were then sequenced on an Illumina Hiseq 2000 platform by Allwegene Technology Inc. (Beijing, China).

### Transcriptome assembly, assessment, and annotation

Raw reads were filtered to remove adaptor-containing, poly-N, and low-quality reads containing more than 50% bases with a Q-value ≤ 5. The remaining high-quality, clean reads were used in subsequent analyses. Transcriptome assembly was performed with Trinity software^50^ with min_kmer_cov set to 2 and all other parameters set to their defaults. Gene functions were annotated by searching public databases, including NR (NCBI non-redundant protein), KOG (eukaryotic Ortholog Groups), Swiss-Prot, GO (Gene Ontology), and KO (KEGG Ortholog), using the BLASTX algorithm with a significance threshold of E-value ≤ 10^−5^. The protein family (Pfam) database was searched against using the HMMER 3.0 program (profile hidden Markov model software)^51^ with an E-value threshold of 10^−5^. Functional categorization by GO terms was performed using Blast2GO software^52^. KEGG (Kyoto Encyclopedia of Genes and Genomes) pathway annotation was carried out using the BLASTX algorithm with an E-value threshold of 10^−5^. Transcription factors were predicted by searching plantTFDB using BLASTX with E-value ≤ 10^−5^.

### Quantification of gene expression levels and differential expression analysis

Gene expression levels were estimated for each sample using RSEM (RNA-Seq by Expectation-Maximization)^53^. The gene expression level was calculated using the FPKM (fragments per kb per Million reads). Differentially-expressed genes (DEGs) were identified and analyzed by the edgeR R package. *P*-values were adjusted using Q-values^54^. GO term enrichment analysis of DEGs was implemented by the GOseq R package based on Wallenius non-central hyper-geometric distribution^55^. KEGG pathway enrichment of differentially expressed genes was performed using KOBAS^56^. The following criteria were used to set the threshold for significant differential expression: Q-value < 0.05 and |log_2_ (fold change)| > 1.

### Weighted Gene Co-expression Network Analysis (WGCNA)

To further screen the DEGs, the R package WGCNA^57^ was used to identify key genes correlated to flower volatiles based on dynamic gene expression changes in different flower tissues. To confirm the analysis accuracy, low-expressed genes with FPKM < 5 in all samples were removed. TO value (topological overlap, unsigned) were calculated for each pair of genes^58–60^, based on which the gene cluster tree was constructed by hierarchical clustering method and further cut into modules by the method of dynamic treecut^57^. Module eigengene (ME) for each module was further obtained by the principal component analysis (PCA). To correlate flower volatiles to modules, contents of flower volatiles in every tissue in developmental or circadian process were listed and made into a matrix. The coefficient factors between the matrix and MEs were calculated, which indicated the correlation level between flower volatile and module. The top three related modules were selected for every flower volatile.

### Quantitative reverse transcription (qRT)-PCR validation of a subset of DEGs

Six flower scent-related unigenes in the development process and eight unigenes related to the circadian process were chosen to validate the RNA-seq results by qRT-PCR. RNA samples used for qRT-PCR were isolated from the same flower tissues used for RNA-seq libraries. cDNA for each sample was synthesized from total RNA using ReverTra Ace qPCR RT Master Mix with gDNA Remover (Toyobo, Japan) according to the instructions. The primers used for qRT-PCR (Supplementary Table S1) were designed with the Primer Premier software (version 5.0). Relative gene expression levels were detected using the SYBR^®^ Green Real-Time PCR Master Mix (Toyobo, Japan) according to the manufacturer’s instructions on a StepOnePlusTM Real-Time PCR System (Life Tech, USA). The quantification of the relative expression of the genes in different times was performed using the delta - delta Ct method as described by Livak and Schmittgen (2001)^61^. All data were expressed as the means ± standard deviation (SD) after normalization. The *Actin* gene was chosen as an internal control. Linear regression analysis was conducted using the fold change values of qRT-PCR and RNA-Seq.

### Data Availability

All transcriptome data was deposited in NCBI Sequence Read Archive with accession number SRP119419.

## Results

### Changes of volatile compound emissions during flower developmental and circadian processes

**Figure 2:**
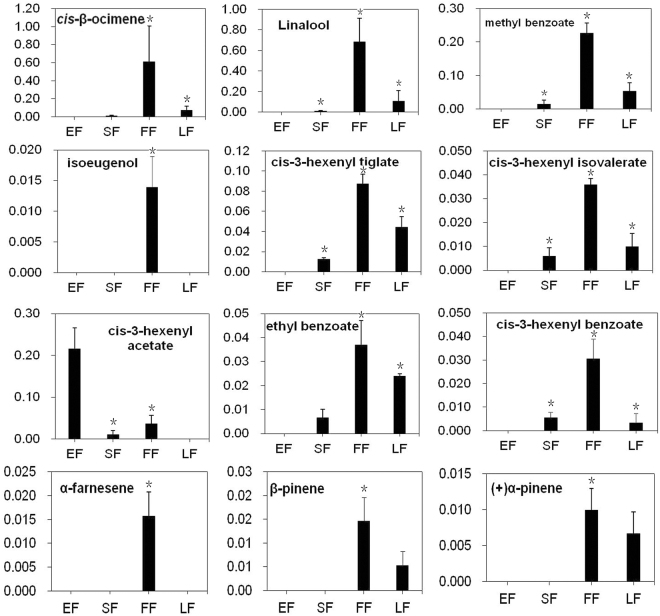
Variation in flower scent compound emission rates across flower developmental stages in *Lilium* ‘Siberia’. Emission rates during FF were the greatest for nearly all floral compounds except cis-3-hexenyl acetate. Means with asterisk (*) are significantly different (Student’s *t* test, P < 0.05).

Volatile compounds emitted from flowers at four flowering stages were sampled by headspace collection and detected by gas chromatograph-mass spectrometer (GC-MS). More than 900 compounds were detected at every stage, respectively. By filtering that accounting for less than 0.01% of the total amount, about 65 compounds were obtained for each flowering stage, respectively. To find changes during flowering process, volatile profiles of the four stages were combined to compare (Supplementary Table S2). By combining the four profiles, a total of 101 volatile compounds were obtained, of which 21 were identified as flower scent compounds of *Lilium* ‘Siberia’ according to descriptions for flower scent^37,62,63^, including cis-β-ocimene, linalool, methyl benzoate, cis-3-hexenyl tiglate, cis-3-hexenyl isovalerate, isoeugenol, cis-3-hexenyl acetate, ethyl benzoate, cis-3-hexenyl benzoate, α-farnesene, β-pinene, (+)-α-pinene, etc. Of the 21 compounds, 19 were found at FF stage, while the EF, SF and LF stages had only 6, 12 and 12, respectively. Moreover, most of the 21 flower scent compounds (18) had the highest emission rate at FF stage, while 3 got their peaks at EF stage, including cis-3-hexenyl acetate (Fig. 2; Supplementary Table S2). These results revealed that the biosynthesis of floral volatiles is controlled developmentally in *Lilium* ‘Siberia’.

**Figure 3:**
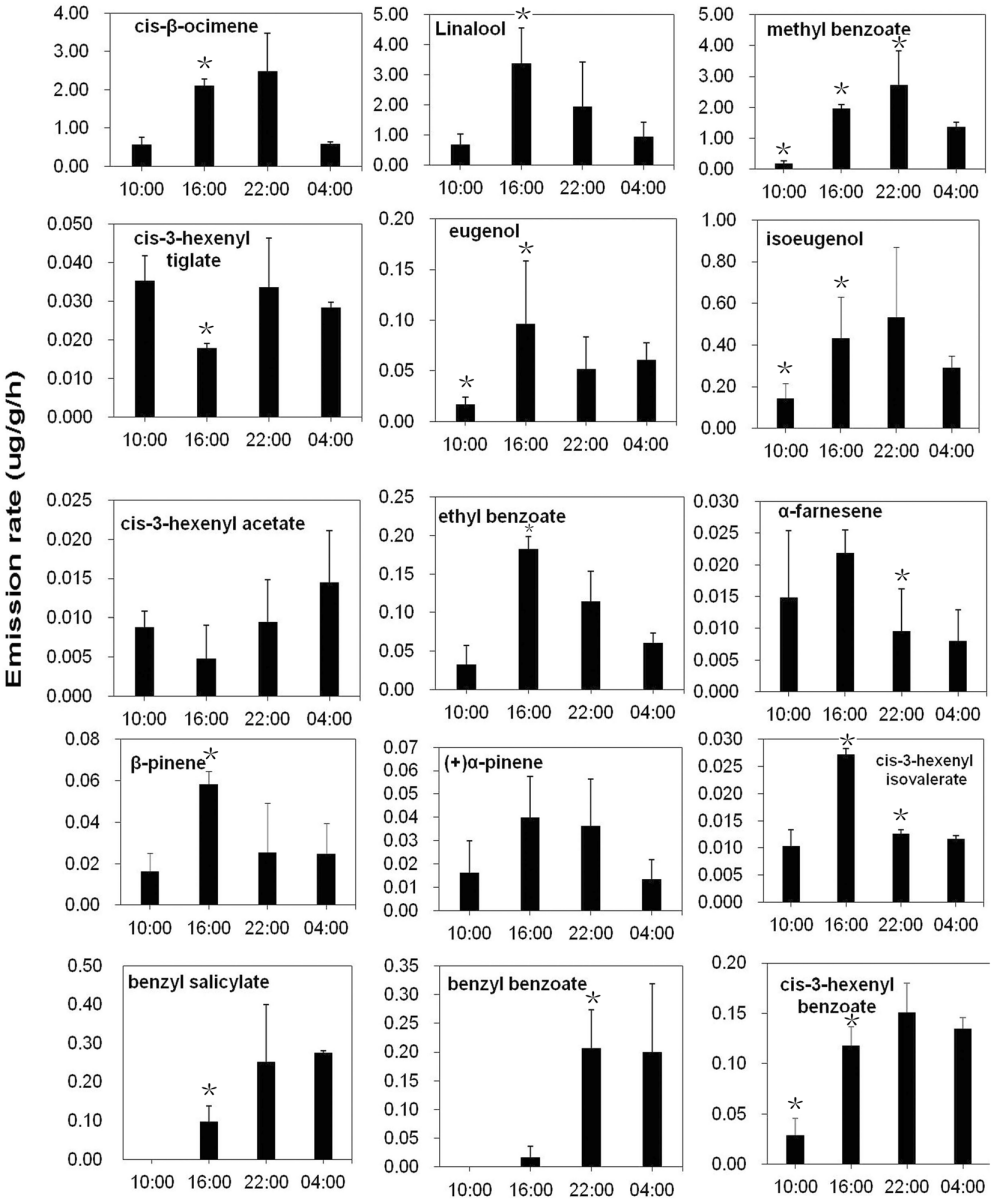
Variation in flower scent compound emission rates across time of day in *Lilium* ‘Siberia’. Floral volatile compound emission rates varied across time of day without uniform pattern detected during flower development. Means with asterisk (*) are significantly different (Student’s *t* test, P < 0.05).

Previous studies have shown rhythmic patterns of scent emission in the flowers of lily ‘Siberia’^37,64^. Flowers at FF stage were used to detect variation in flower volatile compound emission rates at all four circadian time points. Similar to the analysis during development, *cis*-β-ocimene, linalool, and methyl benzoate had the highest the emission rates among all compounds at all four circadian time points. The other nine compounds described above were also detected, in addition to three other compounds: eugenol, benzyl salicylate, and benzyl benzoate. Unlike the pattern seen during flower development, floral volatile compound emission rates varied across time of day (Fig. 3, Supplementary Table S3). However, within the 15 major components in lily floral scents, 8 of items have highest emission rates at 16:00 (including linalool, which represent ~12% of total amount of volatiles of lily, the largest compounds of lily volatiles), 4 for 22:00, 2 for 4:00 and 1 for 10:00. These results supported the previous finding that the generation of floral volatiles occurred in a circadian rhythm in *Lilium* ‘Siberia’^37,64^.

### Sequencing, *de novo* assembly, and gene annotation in RNA-seq of ‘Siberia’ flowers

**Figure 4:**
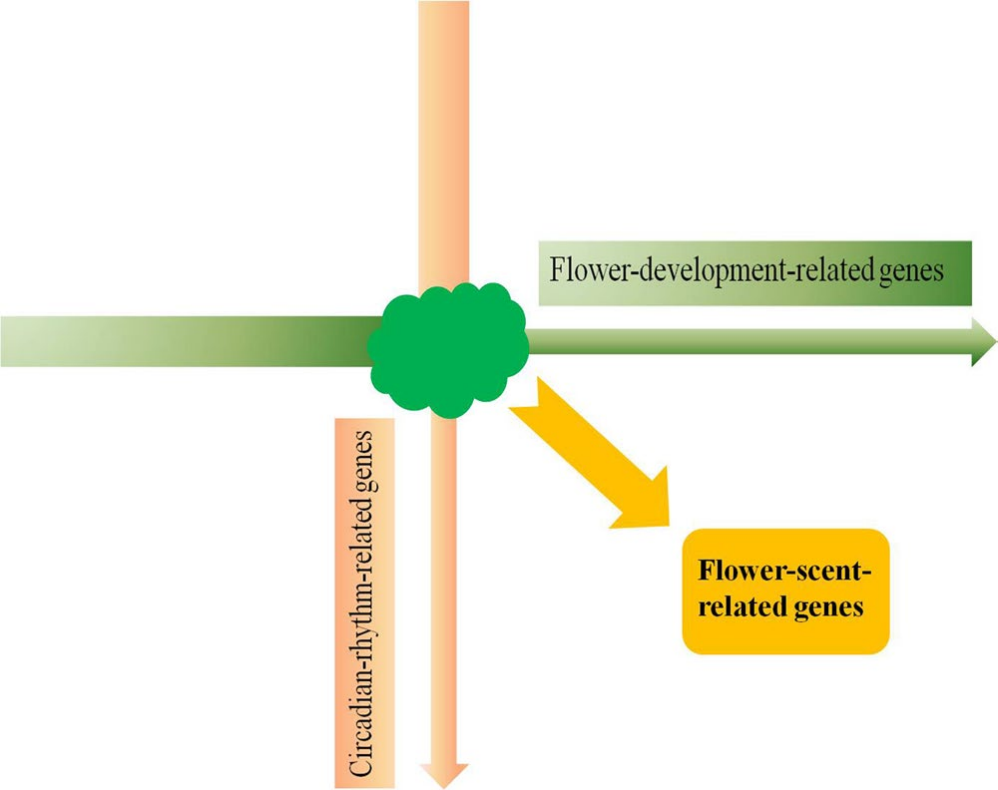
Workflow schematic of two-dimensional (2-D) analysis. GC-MS and RNA-seq are conducted for both flower developmental and circadian rhythmic processes, resulting in common DEGs that are likely responsible for flower scent in *Lilium* ‘Siberia’.

Lily flower tissues from four flower developmental stages, including 12 samples, were first analyzed using RNA-seq, resulting in more than ten thousand DEGs. Since scent-related compound emission rates vary by time of day (Fig. 3)^37^, flower tissues from four time points during the day, including 4 samples, were also analyzed using RNA-seq to allow a 2-D analysis (Fig. 4). Complete sequencing of 16 samples achieved a total of 1.6 billion 150 bp raw reads. After a stringent quality filtering process, 1.6 billion clean reads remained, resulting in 234.38 Gb of clean data with a Q30 percentage (an error probability lower than 0.1%) ranging from 94.98 to 97.58% (Table 1).

**Table 1:** Statistics of transcriptome data of *Lilium* ‘Siberia’.

Samples	Raw reads	Clean reads	Clean base pairs (Gb)	Q20 (%)	Q30 (%)
10:00	113,491,074	106,066,424	17.02	98.37	95.71
16:00	111,181,930	103,908,345	16.68	98.05	94.87
22:00	83,259,410	77,812,532	12.48	98.09	94.98
04:00	97,125,416	90,771,416	14.56	98.15	95.06
EF	312,872,796	292,404,482	44.38	98.96	97.06
SF	291,741,730	272,655,822	41.84	98.86	96.79
FF	285,777,736	267,081,996	40.58	99	97.17
LF	329,562,738	308,002,558	46.76	99.14	97.58

**Figure 5:**
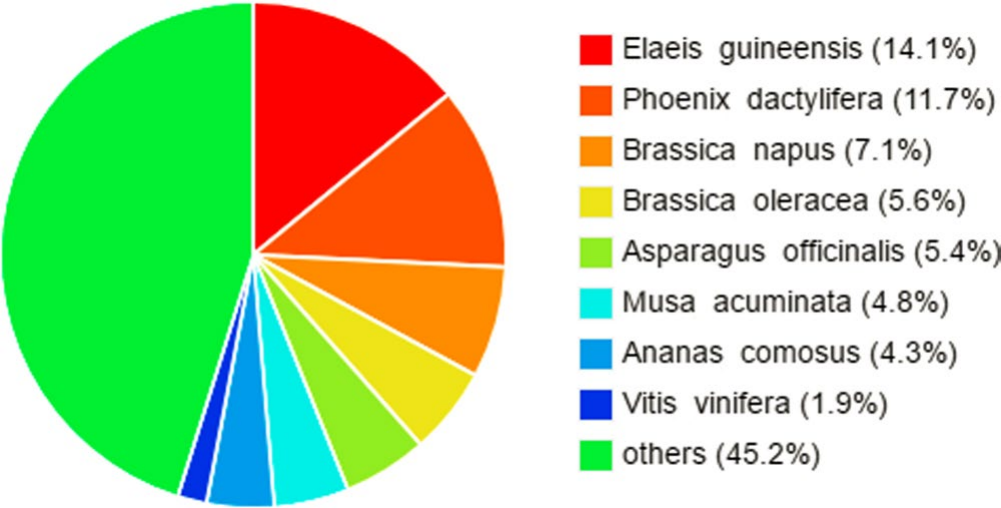
Species distribution of the top BLAST hits by nr annotation for *Lilium* ‘Siberia’. The cut-off values for BLAST search was set at 1.0e^−10^.

For the 2-D analysis, all clean reads required assembly. After assembly, transcripts with equal or more than 10 counts were filtered and finally a total of 118,665 unigenes were obtained, of which N50 was 1,038 bp and the average length was 825 bp. All the unigenes were selected for functional annotation by searching against six public databases (NR, Pfam, KOG, Swiss-prot, KO and GO). A totalof 61,284 (51.64%) were successfully annotated based on similarity to sequences in at least one database (Table 2). Approximately 56,851 unigenes (47.91%) were annotated using sequences from the NR database. With respect to species, 14.05% of sequences had top hits to sequences from *Elaeis guineensis*, followed by *Phoenix dactylifera* (11.72%), *Brassica napus* (7.14%), and *Brassica oleracea* (5.58%) (Fig. 5). A total of 50,256 unigenes (42.35%) were annotated using the KOG databases and grouped into 26 potential functional classifications (Supplementary Figure S1). In addition, 38,202 unigenes (32.19%) were classified into 57 GO functional terms among three main categories: biological processes (BP), cellular components (CC), and molecular functions (MF; Supplementary Figure S2). A total of 13,481 unigenes (11.36%) were mapped to 129 KEGG pathways (Supplementary Table S4), among which several pathways were enriched, including gluconeogenesis (ko00010), tricarboxylic acid (TCA) cycle (ko00020), pentose phosphate pathway (ko00030), fatty acid biosynthesis (ko00061), synthesis and fatty acid degradation (ko00071), and ubiquinone and other terpenoid-quinone biosynthesis (ko00130).

**Table 2:** Statistics of unigenes annotated against public databases.

Annotation Database	Number of annotated unigenes	Percentage of annotated unigenes (%)
NR	56,851	47.91%
KEGG	13,481	11.36%
SwissProt	42,313	35.66%
PFAM	38,828	32.72%
GO	38,202	32.19%
KOG	50,256	42.35%
Annotated in all databases	53,105	44.75%
Annotated in at least one database	61,284	51.64%
Total	118,665	100.00%

### Identification of DEGs and TFs associated with flower scent production

**Figure 6:**
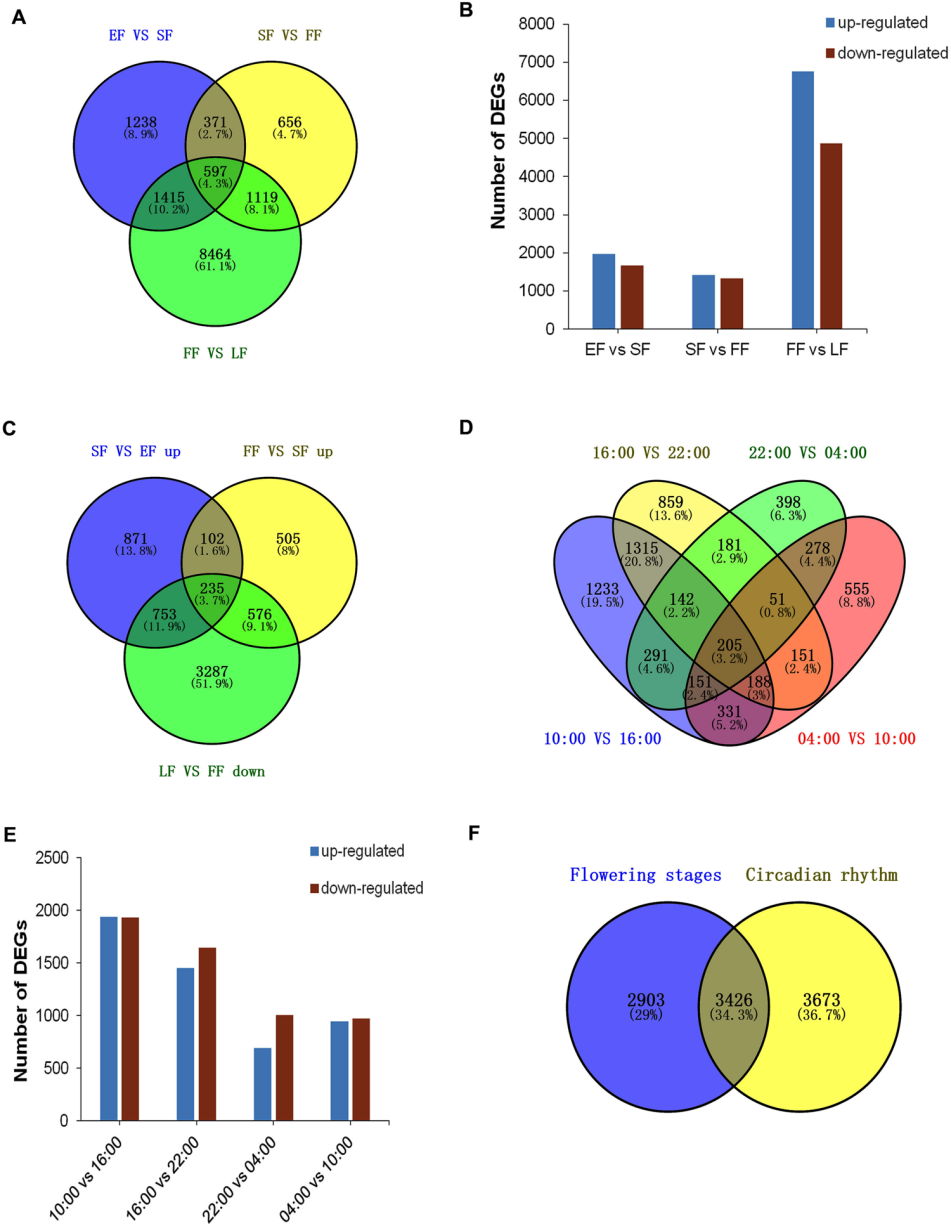
Differentially expressed genes (DEGs) in flower developmental and rhythmic processes in *Lilium* ‘Siberia’. (**A**) Venn diagram of DEGs when comparing successive flower developmental stages from early flowering (EF), semi-flowering (SF), full flowering (FF), and late flowering (LF). (**B**) Graphic presentation of up- and down-regulated DEGs when comparing successive flower developmental stages. (**C**) Venn diagram of up- or down-regulated DEGs corresponding to increases or decreases, respectively, in floral volatile compound emissions when comparing successive flower developmental stages. (**D**) Venn diagram of DEGs in the flower rhythmic process when comparing successive time points. (**E**) Graphic presentation of up- and down-regulated DEGs in circadian rhythmic process; (**F**) Venn diagram of DEGs unique to or shared by the developmental and circadian processes.

DEGs between libraries were determined by pairwise comparisons: EF vs. SF, SF vs. FF, FF vs. LF, 04:00 vs. 10:00, 10:00 vs. 16:00, 16:00 vs. 22:00, and 22:00 vs. 04:00). A total of 13,860 DEGs were obtained for the flower developmental process (Fig. 6A). From EF to SF, 1,961 DEGs were up-regulated, which corresponded to the beginning of flower scent biosynthesis (except for *cis*−3-hexenyl acetate; Fig. 2). From SF to FF, 1,418 DEGs were up-regulated, while from FF to LF, 4,851 DEGs were down-regulated (Fig. 6B), which corresponded to the increase and decrease of flower volatiles, respectively (Fig. 2). After accounting for overlapping DEGs across the three comparisons, a union atlas of 6,329 DEGs was obtained that represented candidate genes (Fig. 6C). During the circadian process, a total of 7,099 DEGs were identified (Fig. 6D,E). Because floral volatile compound emission rates varied by time of day (Fig. 3), all of the DEGs were selected to be candidates.

An atlas with 3,426 DEGs common to both flowering development and circadian rhythm was obtained, representing a large reduction compared to either of the two processes alone (Fig. 6F). GO enrichment analysis was used to classify the potential functions enriched among these DEGs. In the BP category, oxidation-reduction (GO:0055114), lignin biosynthesis (GO:0009809), coumarin biosynthesis (GO:0009805), and cytoplasmic translation (GO:0002181) were among the top function terms. In the CC category, the function terms included cytosol (GO:0005829), plasmodesma (GO:0009506), and plasma membrane (GO:0005886), while the structural constituent of ribosomes (GO:0003735), protein binding (GO:0005515), and transcription factor activity (GO:0003700) were among the top terms of the MF category (Supplementary Figure S3).

**Figure 7:**
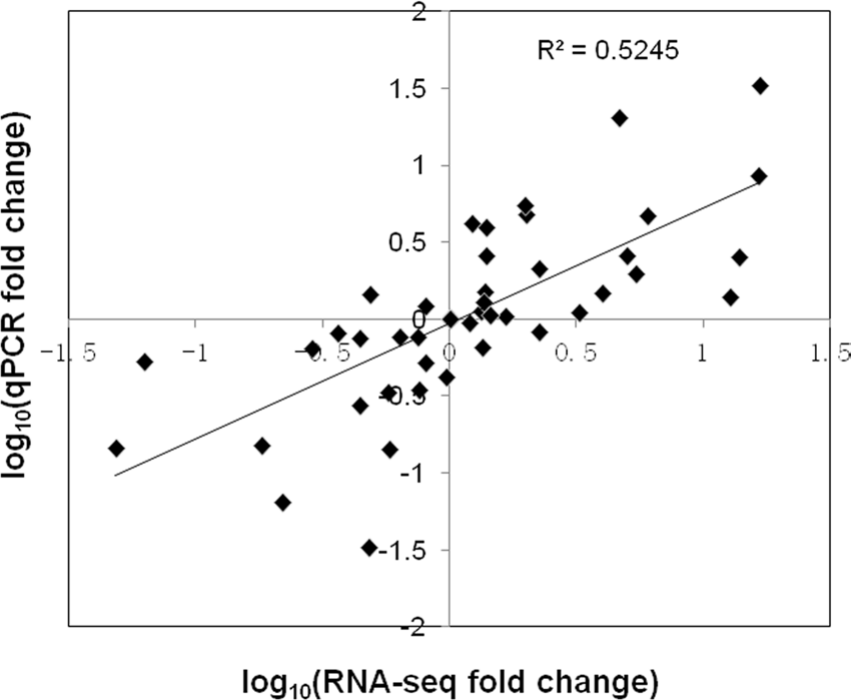
Coefficient analysis between gene expression ratios obtained by RNA-seq and qRT-PCR data. RNA-Seq fold change refers to the ratios of RPKM values of SF, FF, LF to EF or 16:00, 22:00, 04:00 to 10:00 for selected transcripts, while Q-PCR fold change is the relative quantity of SF, FF, LF normalized to expression level of EF, or 16:00, 22:00, 04:00 to that of 10:00. EF: early flowering stage; SF: semi-flowering stage; FF: full flowering stage; LF: late flowering stage.

The expressions of six unigenes in the development process and eight unigenes in the circadian process were verified using qRT-PCR. The qRT-PCR results showed a positive correlation between qRT-PCR and RNA-seq results (Fig. 7, Supplementary Figure S4), indicating good reproducibility of the RNA-seq data in this study.

**Figure 8:**
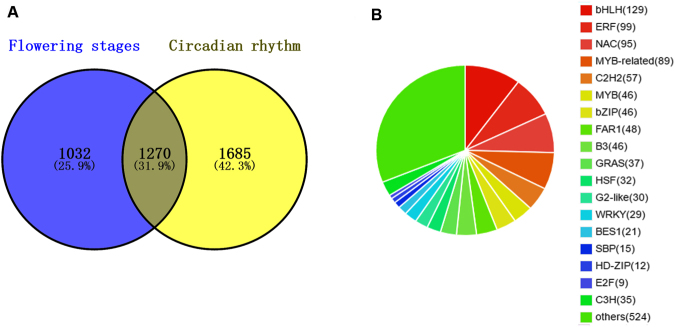
Transcription factors (TFs) identified from the flower developmental and rhythmic processes of *Lilium* ‘Siberia’. (**A**) Venn diagram of differentially-expressed TFs unique to or shared by the development and circadian processes. (**B**) Distribution of differentially-expressed TFs in TF families in *Lilium ‘*Siberia’.

Notably, 24,379 TFs were found to be expressed in ‘Siberia’ flowers. During flower development, there were 2,302 differentially-expressed TFs, 1,270 of which overlapped the 2,955 differentially-expressed TFs identified from the circadian rhythmic analysis (Fig. 8A). The 1,270 differentially-expressed TFs in the combined atlas were classified into 55 families, of which the bHLH family (129 members) accounted for the largest portion, followed by the ERF (99), NAC (95), MYB-related (89), C2H2 (57), MYB (46), and bZIP (46) families (Fig. 8B).

### Analysis of putative genes related to biosynthesis of flower scent compounds

To detail the biosynthesis of flower scent, the genes were filtered for those believed to be involved in the putative flower scent biosynthesis pathway for *Lilium* ‘Siberia’, which was determined from previous research^63,65^.

For terpenoids, eight unigenes encoding enzymes involved in the MEP pathway were identified in present transcriptome, including one each of 1-deoxy-Dxylulose 5-phosphate synthase (DXS) genes, 1-deoxy-D-xylulose 5-phosphate reductoisomerase (DXR) gene, 2-C-methyl-D-erythritol 4-phosphate cytidylyltransferase (MCT) gene, 4-(cytidine 5′-diphospho)-2-C-methyl-D-erythritol kinase (CMK) gene, two 2-C-methyl-Derythritol-2,4-cyclodiphosphate synthase (MDS) genes, and two 4-hydroxy-3-methylbut-2-en-1-yl diphosphate reductase (HDR) genes. Six unigenes were identified for encoding enzymes involved in the MVA pathway, including two acetyl-CoA acetyltransferase (AACT) genes, one hydroxymethylglutaryl-CoA synthase (HMGS) gene, two hydroxymethylglutaryl-CoA reductase (HMGR) genes and one mevalonate kinase (MK) gene (Supplementary Figure S5, Supplementary Table S5). Expressions of major unigenes above presented a concomitant change with flower scent during the flower development, while it was complex during the circadian process. After formation of IPP and DMAPP by the above enzymes, GPP and FPP were generated by GPP synthase (GPPS) and FPP synthase (FPPS), the precursors of mono- and sesqui-terpenes, respectively^66^. One GPPS unigene and two FPPS unigenes were identified from the current assembly transcriptome. GPPS unigene had a similar expression trend with the emission of terpenes, while FPPS unigenes did not, which was consistent with foundings in *Hedychium coronarium*^65^. An array of monoterpenes and sesquiterpenes are generated through the action of TPSs, which directly determine product specificity^67^. Nineteen TPS unigenes were identified in the current transcriptome (Supplementary Figure S5, Supplementary Table S5), including unigenes for monoterpens linalool_synthase (LIS), (E)-beta-ocimene synthase, myrcene synthase, and sesquiterpenes (3 S,6E)-nerolidol synthase (NES). The majority of TPS unigenes presented expression changes correlated to terpenoids biosynthesis during flower development and circadian rhythm process.

The first committed step in benzenoid biosynthesis was catalyzed by phenylalanine ammonialyase (PAL), which deaminated phenylalanine to cinnamic acid^68^. Seven PAL unigenes existed in the current transcriptome and the expressions were consistent with benzenoid emission during flower development but nearly constant during the circadian process. The formation of benzenoids from cinnamic acid proceeds via β-oxidative pathway and non β-oxidative pathway^18^. The β-oxidative pathway in petunia flowers needed four reactions catalyzed by three enzymes, including cinnamate:CoA ligase/acylactivating enzyme (CNL/AAE), cinnamoyl-CoA hydratasedehydrogenase (CHD) and 3-ketoacyl CoA thiolase (KAT)^69–72^. Only two KATs were found in present transcriptome, of which expressions escalated slightly following flower development (Supplementary Figure S6). To non-β-oxidative pathway, NAD-dependent benzaldehyde dehydrogenase (BALD) accounted for the oxidation of benzaldehyde into benzoic acid^73^. Three BALD homologs were identified, but the expressions did not show correlation with the emission of benzenoids. For the final step for benzenoid biosynthesis, twenty-two S-adenosyl-L-methionine-dependent methyltransferase (SAM-Mtases) unigenes were identified in the transcriptome (Supplementary Figure S6, Supplementary Table S5), several of which could be identified as that for benzoic acid carboxyl methyltransferase (BAMT), benzoic acid/salicylic acid carboxyl methyltransferases (BSMT), salicylic acid carboxyl methyltransferase (SAMT), etc. Most of the SAM-Mtases unigenes presented expression trends correlated with benzenoid biosynthesis during flower development.

In biosynthesis pathway of volatile fatty acid derivatives, four LOX unigenes were identified from the transcriptome (Supplementary Table S5). Expressions of LOX unigenes had the highest level at the EF stages and decreased following the flower development (Supplementary Figure S7). Similar pattern could be found to hydroperoxide lyase unigene, which was responsible for converting 9- and 13-hydroperoxy intermediates into volatile C6 and C9 aldehydes. Fourteen unigenes were identified from the transcriptome for alcohol dehydrogenases, which could convert the C6 and C9 aldehydes to volatile alcohols^25^. Moreover, one unigene for stearoyl-acyl carrier protein 9-desaturase was identified (Supplementary Table S5), which could desaturate fatty acids into produce 18:1Δ9 (*ω*-9) fatty acid intermediate, from which 9-alkenes could be derived^74^.

### Weighted gene co-expression network analysis (WGCNA) of DEGs

We used WGCNA to further filter the DEGs by correlating them with emission level changes of floral volatile compounds. A total of 26,049 genes, including all the DEGs, were clustered into 43 modules by WGCNA. All genes with GO annotations in the modules are listed in Supplementary Table S6. A correlation map between modules and flower scent compounds was generated (Supplementary Figure S8), from which the top three modules were selected for each compound. By this method, we identified 12 modules related to the top 15 floral volatiles of *Lilium* ‘Siberia’ (Table 3). Four modules were correlated with both monoterpene and sesquiterpenes, including brown, pale-turquoise, saddle-brown and dark-orange. In phenylpropanoids, methyl benzoate, ethyl benzoate as well as (iso)eugenol and eugenol all correlated with pale-turquoise and brown modules, while benzyl salicylate and benzyl benzoate shared the same modules of yellow, dark-orange, and brown. As fatty acid derivatives, *cis*−3-hexenyl tiglate and *cis*−3-hexenyl isovalerate both correlated with dark-turquoise, plum1, and dark-orange modules. As expected, *cis*−3-hexenyl benzoate shared the same modules with the phenylpropanoid methyl benzoate. By this analysis, DEGs were further correlated to flower scent compounds through modules.

**Table 3:** Modules identified by weighted gene co-expression network analysis (WGCNA) analysis responsible for flower volatiles in *Lilium* ‘Siberia’. For every flower scent volatile, three modules with top high coefficient factors from all the 43 modules were selected, respectively.

	Module 1	Module 2	Module 3
*cis*-β-ocimene	brown	pale-turquoise	dark-orange
linalool	pale-turquoise	saddle-brown	brown
α-farnesene	blue	pale-turquoise	dark-orange
β-pinene	pale-turquoise	saddle-brown	brown
(+)-α-pinene	pale-turquoise	saddle-brown	brown
methyl benzoate	brown	pale-turquoise	dark-orange
ethyl benzoate	pale-turquoise	brown	light-steel-blue1
benzyl salicylate	yellow	dark-orange	brown
benzyl benzoate	brown	dark-orange	yellow
isoeugenol	pale-turquoise	saddle-brown	brown
eugenol	pale-turquoise	saddle-brown	brown
*cis*-3-hexenyl tiglate	dark-turquoise	plum1	dark-orange
*cis*-3-hexenyl isovalerate	dark-turquoise	plum1	dark-orange
*cis*-3-hexenyl acetate	dark-grey	green-yellow	pink
*cis*-3-hexenyl benzoate	brown	pale-turquoise	dark-orange

### Identification of putative genes regulating biosynthesis of flower scent compounds

In order to narrow the range of candidate genes, we then searched the 3,434 DEGs common to flower development and circadian rhythm data sets against every WGCNA module. A total of 1,210 DEGs partitioned into the 12 modules selected above. Blue (241) contained the largest number of DEGs, followed by dark-orange (176), dark-turquoise (170), brown (138), yellow (136), light-steel-blue1 (75), pink (66), dark-grey (54), pale-turquoise (44), plum1 (38), green-yellow (38), and saddle-brown (34). We then generated heat maps of gene expression for each module (Supplementary Figure S9). For cis-3-hexenyl acetate, which showed an emission pattern different from the other 14 compounds, DEGs were obtained individually from the two processes and then categorized into the related modules: dark-grey (84), pink (84), and green-yellow (70). All DEGs with annotations are listed in Supplementary Table S7. We also screened the 1,273 differentially-expressed TFs that overlapped between development and circadian processes against the 12 modules. A total of 419 TFs partitioned into modules, including 74 TFs in the dark-turquoise module. The remaining TFs were distributed as follows: blue (70), yellow (39), brown (37), light-steel-blue1 (34), dark-orange (32), pink (32), dark-grey (26), pale-turquoise (21), saddle-brown (20), plum1 (17) and green-yellow (17). For *cis*−3-hexenyl acetate, 43, 40 and 30 TFs partitioned into pink, dark-grey and green-yellow modules, respectively. All the TFs with annotations are listed in Supplementary Table S7.

**Figure 9:**
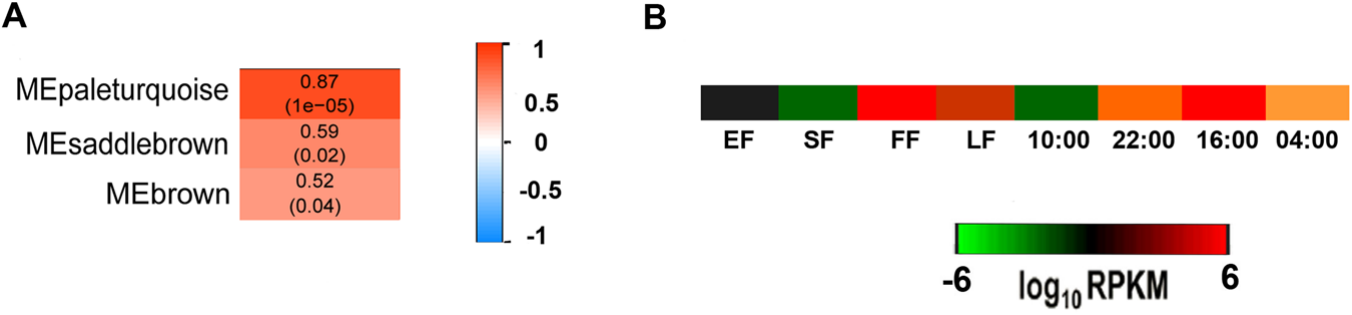
Linalool-related modules and expression changes of *LIS*. (**A**) Top three modules related to linalool according to the association heat map between modules and flower volatile compounds from WGCNA analysis. The three modules own top high coefficient factors among all the 43 modules. (**B**) *LIS* expression changes across flower developmental stages and time of day in the brown module. EF: early flowering stage; SF: semi-flowering stage; FF: full flowering stage; LF: late flowering stage. LIS: linalool synthase.

Four common modules were associated with monoterpenes and sesquiterpenes based on the WGCNA results. A key gene in the last step for linalool biosynthesis, *linalool synthase* (LIS^75^), was found in the brown module (Fig. 9) together with an *alcohol acetyl transferase* (*AAT*) unigene (DN245787_c1_g3_i2) involved in monoterpene alcohol biosynthesis^76,77^ and the *(3 S*,*6E)-nerolidol synthase* unigene (DN251718_c0_g2_i1) involved in both nerolidol and linalool biosynthesis^78^, which further confirmed the involvement of this module in terpenoid biosynthesis. From this module, we could find other candidate unigenes, such those encoding auxin-responsive protein (DN244632_c1_g2_i1), acyl transferase (DN226095_c0_g2_i1), gibberellin-regulated proteins (DN228276_c0_g1_i1, DN232815_c0_g1_i1), gibberellin 2-beta-dioxygenase (DN247305_c0_g1_i2, DN250921_c0_g2_i3), and adenosine triphosphate–binding cassette (ABC) transporter (DN253634_c1_g2_i1). Thirty-seven TFs were involved in brown module, of which three belonged to the MYB family and five belonged to the bHLH family.

Phenylpropanoids were related to seven modules in the WGCNA results. In the pale-turquoise module, unigenes encoding several known phenylpropanoid-related enzymes, including benzyl alcohol O-benzoyltransferase (BEBT, DN240712_c1_g4_i1), cinnamoyl-CoA reductase (DN236463_c1_g1_i1), anthocyanin 5-aromatic acyltransferase (DN244249_c0_g1_i2), and 4-coumarate–CoA ligase (DN248980_c2_g11_i1) were found, verifying role of this module in phenylpropanoid biosynthesis. Unigenes encoding other phenylpropanoid-related enzymes were found in the saddle-brown module, including for SAM-Mtases (DN87519_c0_g2_i1), phosphoenolpyruvate translocator in the shikimate pathway (DN248494_c1_g3_i1), arogenate dehydratase (DN252205_c0_g1_i1, DN252205_c0_g3_i1), together with an ABC transporter (DN235235_c0_g1_i1), and an ethylene-responsive transcription factor (DN242444_c0_g1_i1). Forty-one TF genes were involved in the two modules, including two encoding MYB TFs.

A total of eight modules were associated with fatty acid derivatives based on the WGCNA results. In the green-yellow module, two unigenes involved in the LOX pathway were found: one encoding lipoxygenase (DN253294_c0_g7_i5) and one encoding fatty acid hydroxylase (DN247380_c2_g3_i2). Seventeen TF genes were identified in this module, including two MYBs, three bHLHs, and two B3s. In the dark-turquoise module, nine unigenes related to fatty acid derivatives were found, including three for acyl-[acyl-carrier-protein] desaturase (DN248341_c1_g1_i1, DN219892_c0_g3_i1, DN253694_c7_g2_i1), one for a fatty acid transporter (DN233787_c1_g2_i2), one for both fatty acyl-CoA reductase and aldehyde dehydrogenase (NAD; DN240253_c0_g2_i2), three for fatty-acyl-CoA synthase (DN248980_c2_g14_i1, DN248980_c2_g16_i1, DN248980_c2_g1_i1) and one for 2-alkenal reductase (DN252291_c1_g5_i1). In addition, four unigenes for auxin-responsive proteins (DN242854_c1_g2_i1, DN242854_c1_g3_i1, DN250599_c1_g1_i3, DN252722_c0_g9_i1) were also included. This module contained 73 TF genes, including nine MYBs, 15 bHLHs, five B3s, and six C2H2s. By combining results of 2-D and WGCNA analyses, we decreased the candidate number and several genes (TFs) were identified for the three classes of flower scent compounds, respectively.

## Discussion

Corresponding to its role in plant fertilization, flower scent production presents an evident change during flower development^18,79–81^. However, other processes are involved floral development, such as petal elongation, flower color and flower senescence, which interfere with identifying candidate genes for flower scent by RNA-seq. Circadian clock exerts rhythmic emission of floral scent in numerous plant species^35,79,82–85^, including lily^37,64^, which provides another way to screen the DEGs for flower scent. In the present study, a 2-D analysis was conducted to eliminate irrelative DEGs. Since floral scent varies diurnally, we conducted GC-MS and RNA-seq analyses across both flower developmental stages and time of day. The GC-MS results confirmed the developmental and circadian changes in flower scent production in lily. A total of 1.6 billion clean reads were generated from RNA-seq analyses, and 118,665 unigenes were obtained after assembling all reads together. Finally, up to 61,248 unigenes were annotated. To our best knowledge, this is the largest transcriptomic data set produced in *Lilium*. The data not only provide resources for gene isolation in flower scent biosynthesis but also resources for insight into flower development and circadian rhythms of *Lilium* species.

Numerous DEGs were found in both floral developmental and rhythmic processes. A total of 6,329 DEGs were identified in flower development, while 7,099 were found from the circadian rhythm. In the 2-D analysis, 3,426 candidate genes overlapped between the two processes. We therefore eliminated DEGs which might be involved in interfering processes, such as petal elongation, flower color, and flower senescence. By GO and KEGG enrichment, we found that the DEG functions were enriched to several pathways related to secondary metabolic pathways, including oxidation-reduction, lignin biosynthesis, and coumarin biosynthesis. The cellular component and molecular function enrichments, such as protein binding and transcription factor activity, suggested that some of the DEGs functioned in pathway regulation. TFs have been shown to be involved in coordinated regulation of entire scent biosynthetic networks^86,87^. In the study, a total of 1,270 identified TFs corresponded to flower volatile changes in the two processes. These were classified into 55 TF families, in which the bHLH family, with 129 members, represented the most abundant. In addition, 46 MYB TFs were identified.

WGCNA has been used as a powerful tool to identify key genes associated with specific biological processes or phenotypes, such as seed germination^88^, response to drought stress^89^, GA_3_-induced fruit setting^90^, and fruit acidity^91^. In the present study, a total of 12 modules were associated with the 15 flower volatile compounds identified by GC-MS. Similar modules were associated with homogenous compounds, indicating that DEGs with similar functions were clustered into similar modules across various tissues.

The regulation of terpenoid biosynthesis is complex, and the pathway fluxes are mostly controlled at the transcript level^92^. A unigene for lily *LIS* was found in the brown module, together with two other terpenoid-related unigenes: *NES* and *AAT*. Nerolidol synthase (NES) generates nerolidol from FPP; it is bifunctional in some plants and can also produce linalool from GPP^78^. In plants, LIS and NES usually have very similar catalytic properties but synthesize linalool and nerolidol as specific products from GPP and FPP, respectively, because of their compartmental locations in the cell^93^. The clustering of the two genes in the same module suggested their coordinated regulation. AAT plays an important role in modification of floral volatiles by transferring various alcohols to their acetate esters^63,64^, including geraniol, citronellol, nerol, phenylethyl alcohol, cis-3-hexen-1-ol, and 1-octanol^80,94^. Other unigenes in this module included those encoding auxin-responsive proteins, acyl transferases, gibberellin-regulated proteins, gibberellin 2-beta-dioxygenases, and ABC transporters.

Hormones are known to regulate volatile production in flowers. Emission of floral volatiles is reduced after pollination or exogenous ethylene treatment, paralleling the reduction of scent-related gene expression levels in *P*. *hybrid* and *Lathyrus odoratus*^95–98^. Gibberellin (GA) is also found to negatively regulate scent production in petunia flowers; this effect is exerted through transcriptional/post-transcriptional down-regulation of regulatory and biosynthetic scent-related genes^99^. A correlation between auxin and flower volatile production is also possible. In transgenic petunia plants, the suppression of *BPBT*, the gene responsible for benzyl benzoate biosynthesis, results in increased auxin transport^100^. Our results suggested that both auxin and gibberellin may affect flower scent production in lily.

An ABC-transporter-dependent mechanism is also involved in floral volatile emission; down-regulation of an ABC transporter gene *PhABCG* results in decreased emission of volatiles and increased toxic accumulation levels in the plasma membrane of *P*. *hybrid*^101^. The ABC transporter may also be involved in the metabolic ‘crosstalk’ between the MEP and MVA pathways^102–105^. In this study, the ABC transporter unigene identified in the brown module may be related to the transport of intermediates or volatiles in the pathways.

Only two TFs have been isolated for transcriptional regulation of the terpenoids pathways. One was a basic helix-loop-helix type, MYC2, activating expressions of two sesquiterpene synthase genes *TPS21* and *TPS11*^35^ and one in AP2/ERF family, CitERF71, up-regulating the expression of monoterpene synthase gene *CitTPS16* and consequently controlling the production of E-geraniol in *Citrus* fruit^36^. Besides, some master TFs may act upstream of multiple metabolic pathways, thus regulating both the terpenoid and phenylpropanoid pathways, such as a MYB TF in Arabidopsis^106^. In the present study, 37 TFs were found in the brown module, including five bHLHs, seven ERFs and three MYBs, which may be involved in the regulation of flower scent biosynthesis.

Multiple branches occur in the phenylpropanoid/benzenoid pathways downstream of phenylalanine, and the compound diversity is further increased by modifications, such as methylation, hydroxylation, and acetylation, to scent precursors^63^. A unigene encoding a sequence similar to SAM-Mtases, which are key enzymes in phenylpropanoid and flavonoid metabolic pathways^107^, was included in the saddle-brown module. Multiple SAM-Mtases are involved in the biosynthesis of phenylpropanoid compounds, including BAMT and BSMT for methyl benzoate^95,108^, POMT^19^, OOMT for DMT and TMB^20,21^, (iso)eugenol O-methyltransferase (IEMT) for (iso)eugenol^109^, and SAMT for methyl salicylate^110^. Another modification enzyme unigene, *BEBT*, was found in the pale-turquoise module. BEBT is a member of the BAHD superfamily of acyltransferases^111^, which catalyzes the formation of benzyl benzoate by transferring the benzoyl group to benzyl alcohol^112^. Other members of the BAHD superfamily of acyltransferases are also involved in the biosynthesis of phenylpropanoids and catalyze the biosynthesis of acetylated scent compounds, such as benzyl acetate in *Clarkia*^113^, benzoyl benzoate in *Clarkia* and *Petunia*^18,100,112^, and phenylethyl benzoate in *Petunia* flowers^18,100^. Cinnamoyl-CoA reductase, a key enzyme in lignin biosynthesis^114^, is also included in the pale-turquoise module, which may be involved in the formation of (iso)eugenol and methyl(iso)eugenol because they share the same initial biosynthetic steps up to the coniferyl alcohol stage^63^. This module also included unigenes for an ABC transporter, an ethylene-responsive transcription factor, and an arogenate dehydratase, which were also suggested to be involved in phenylpropanoid pathways. Six TFs have been identified to regulate gene expressions for phenylpropanoids/benzenoids production^27–30,33,34^. Notably, all of them are in the MYB family, suggesting important roles for this family in flower scent production. From the two modules, two MYB TFs were found, which may function in the regulation of phenylpropanoid biosynthesis.

In the present study, unigenes for linoleate lipoxygenase and fatty acid hydroxylase were found in the green-yellow module, suggesting the involvement of members of that module in the LOX pathway for production of volatile fatty acid derivatives. Other unigenes related to fatty acid derivatives were found in the dark-turquoise module, including those encoding a fatty acid transporter, fatty acyl-CoA reductase, and fatty-acyl-CoA synthase. Unigenes were also found in this module encoding ACP desaturase and 2-alkenal reductase, which are both required for alkene production^74^. In addition, three unigenes encoding auxin-responsive proteins were present in the dark-turquoise module, suggesting that auxin may function in the LOX pathway. A total of 90 TFs were involved in the two modules with 11 MYBs, 18 bHLHs, seven B3s, and seven C2H2s. The regulation of volatile fatty acid derivative biosynthesis has not been yet been elucidated, but the TFs identified in this study may play a role in their production.

## Conclusion

In present study, we conducted an approach combining 2-D analysis and WGCNA method to explore candidate genes involved in regulation of flower scent production. The 2-D analysis produced a core DEG atlas with 3,426 DEGs as initial candidates likely responsible for the flower scent production, including 1,270 TF genes. Multiple unigenes of essential enzymes for flower scent production such as TPSs or SAMTs, were identified. By further associating DEGs of the 2-D analysis with 15 flower volatile compound emissions, a total of 168 TFs, including 16 MYBs, were identified as candidates for regulation of all the three flower volatile classes. In addition, it revealed that auxin, gibberellin may play important role in regulating flower scent production. Our findings provide a foundation that will enable characterization of the molecular mechanisms involved in floral scent formation and regulation in plants. In addition, the results reveal that 2-D analysis combined with WGCNA allowed us to narrow down candidate genes involved in regulation of flower scent production and is a promising strategy to study biosynthesis of secondary metabolites in plants.

## Electronic supplementary material

Supplementary Information

Dataset 1
