# Supplementary material for: Two-dimensional analysis provides molecular insight into flower scent of Lilium ‘Siberia’
Source: Sci Rep. 2018 Mar 29;8:5352. doi: 10.1038/s41598-018-23588-9 (PMC5876372; doi:10.1038/s41598-018-23588-9)
Supplement: Supplementary file 1 — Supplementary Information [file 41598_2018_23588_MOESM1_ESM.docx]

**SUPPLEMENTARY INFO**

**Two-dimensional Analysis Provides Molecular Insight into Flower Scent of *Lilium* ‘Siberia’**

Shaochuan Shi^1#^, Guangyou Duan^2#^, Dandan Li^1^, Jie Wu^1^, Xintong Liu^1^, Bo Hong^1^, Mingfang Yi^1*^ and Zhao Zhang^1*^

^1^Beijing Key Laboratory of Development and Quality Control of Ornamental Crops, Department of Ornamental Horticulture, China Agricultural University, Beijing, China

^2^Energy Plant Research Center, School of Life Sciences, Qilu Normal University, Jinan, China

^#^These authors contributed equally to this work.

*Correspondence to:*

Zhao Zhang

Department of Ornamental Horticulture, China Agricultural University, Yuanmingyuan Xilu 2, Beijing 100193, China.

Tel. 0086-10-62733603

e-mail: zhangzhao@cau.edu.cn

Mingfang Yi

Department of Ornamental Horticulture, China Agricultural University, Yuanmingyuan Xilu 2, Beijing 100193, China.

Tel. 0086-10-62733817

e-mail: ymfang@cau.edu.cn

**Supplementary Figures**

**Figure S1. KOG classification of the unigenes.** A total of 50,256 unigenes were annotated against KOG database and classed into 26 functional categories.

**Figure S2. GO classification of unigenes.** The GO terms are summarized into three main categories: biological process, cellular component and molecular function.

**Figure S3. GO classification of DEGs.** The GO terms are summarized into three main categories: biological process, cellular component and molecular function.

**Figure S4. Q-PCR validation of selected unigenes expression across flowering stages and time of the day.** Expression levels of selected transcripts measured by Q-PCR and RNA-Seq are showed in the same histograms. Black columns indicate relative gene expression levels detected by Q-PCR (left y-axis; normalized units). Grey columns represent expression determined by RNA-Seq in RPKM units (right y-axis). AAT , alcohol acetyl transferase; LIS,  linalool synthase; IEMT, (iso)eugenol o-methyltransferase; BEBT, benzyl alcohol benzoyl transferase; MYS, myrcene sythase; OCS, *cis*-β-ocimene synthase; GPPS, geranyl pyrophosphate synthase; BSMT, benzoic acid/salicylic acid carboxyl methyltransferase; BAMT, benzoic acid carboxyl methyltransferase.

**Figure S5. Expression patterns of genes encoding enzymes possibly involved in terpenoids biosynthesis of *Lilium '*Siberia'**. The abbreviated name of enzyme in each catalytic step is showed in bold. Gene expression levels (log_10_ RPKM) in four flower developmental stages and four circadian time points in *Lilium* ‘Siberia’ (EF: early-flowering; SF: semi-flowering; FF: Full-flowering; LF: Late-flowering) are represented by color gradation. Gene expression with RPKM = 0 was set to -4 after log_10_ transformation. Genes with more than one homology are represented by equal colored horizontal stripe and are termed from top to bottom in Arabic numerical order. DXS, deoxy-Dxylulose 5-phosphate synthase; DXR, 1-deoxy-D-xylulose 5-phosphate reductoisomerase; MCT, 2-C-methyl-D-erythritol 4-phosphate cytidylyltransferase; CMK, 4-(cytidine 5’-diphospho)-2-C-methyl-D-erythritol kinase; MDS, 2-C-methyl-Derythritol-2,4-cyclodiphosphate synthase; HDS, 4-hydroxy-3-methylbut-2-en-1-yl diphosphate synthase; HDR, 4-hydroxy-3-methylbut-2-en-1-yl diphosphate reductase; AACT, acetyl-CoA acetyltransferase; HMGS, hydroxymethylglutaryl-CoA synthase; HMGR, hydroxymethylglutaryl-CoA reductase; MK, mevalonate kinase; PMK, phosphomevalonate kinase; MDC, mevalonate diphosphate decarboxylase; GPPS, geranyl pyrophosphate synthase; FPPS, farnesyl pyrophosphate synthase; LIS, linalool_synthase; NES, (3S,6E)-nerolidol synthase; TPS, terpene synthase.

**Figure S6. Expression patterns of genes encoding enzymes possibly involved in benzenoids biosynthesis of *Lilium '*Siberia'**. The abbreviated name of enzyme in each catalytic step is showed in bold. Gene expression levels (log_10_ RPKM) in four flower developmental stages and four circadian time points in *Lilium* ‘Siberia’ (EF: early-flowering; SF: semi-flowering; FF: Full-flowering; LF: Late-flowering) are represented by color gradation. Gene expression with RPKM = 0 was set to -4 after log_10_ transformation. Genes with more than one homology are represented by equal colored horizontal stripe and are termed from top to bottom in Arabic numerical order. PAL, phenylalanine ammonialyase; PXA, peroxisomal ATP-binding cassette transporters; CNL, cinnamate:CoA ligase/acylactivating enzyme; CHD, cinnamoyl-CoA hydratasedehydrogenase; KAT, 3-ketoacyl CoA thiolase; BALD, NAD-dependent benzaldehyde dehydrogenase; BAMT, benzoic acid carboxyl methyltransferase; BSMT, benzoic acid/salicylic acid carboxyl methyltransferases; SAMT, salicylic acid carboxyl methyltransferase; BEBT, benzyl alcohol O-benzoyltransferase; EGS, eugenol synthase; IGS, (iso)eugenol synthase.

**Figure S7. Expression patterns of genes encoding enzymes possibly involved in fatty acid derivatives biosynthesis of *Lilium '*Siberia'**. The abbreviated name of enzyme in each catalytic step is showed in bold. Gene expression levels (log_10_ RPKM) in four flower developmental stages and four circadian time points in *Lilium* ‘Siberia’ (EF: early-flowering; SF: semi-flowering; FF: Full-flowering; LF: Late-flowering) are represented by color gradation. Gene expression with RPKM = 0 was set to -4 after log_10_ transformation. Genes with more than one homology are represented by equal colored horizontal stripe and are termed from top to bottom in Arabic numerical order. LOX, lipoxygenase; ACP desaturase, stearoyl-acyl carrier protein desaturase.

**Figure S8. Associations between module and flower volatiles by WGCNA analysis.** Each row corresponds to a module eigengene, column to a trait. Each cell contains the corresponding correlation and p-value. The table is color-coded by correlation according to the color legend.

**Figure S9. Heatmap of the gene expressions in the12 modules.**

**Supplementary Figure 1**


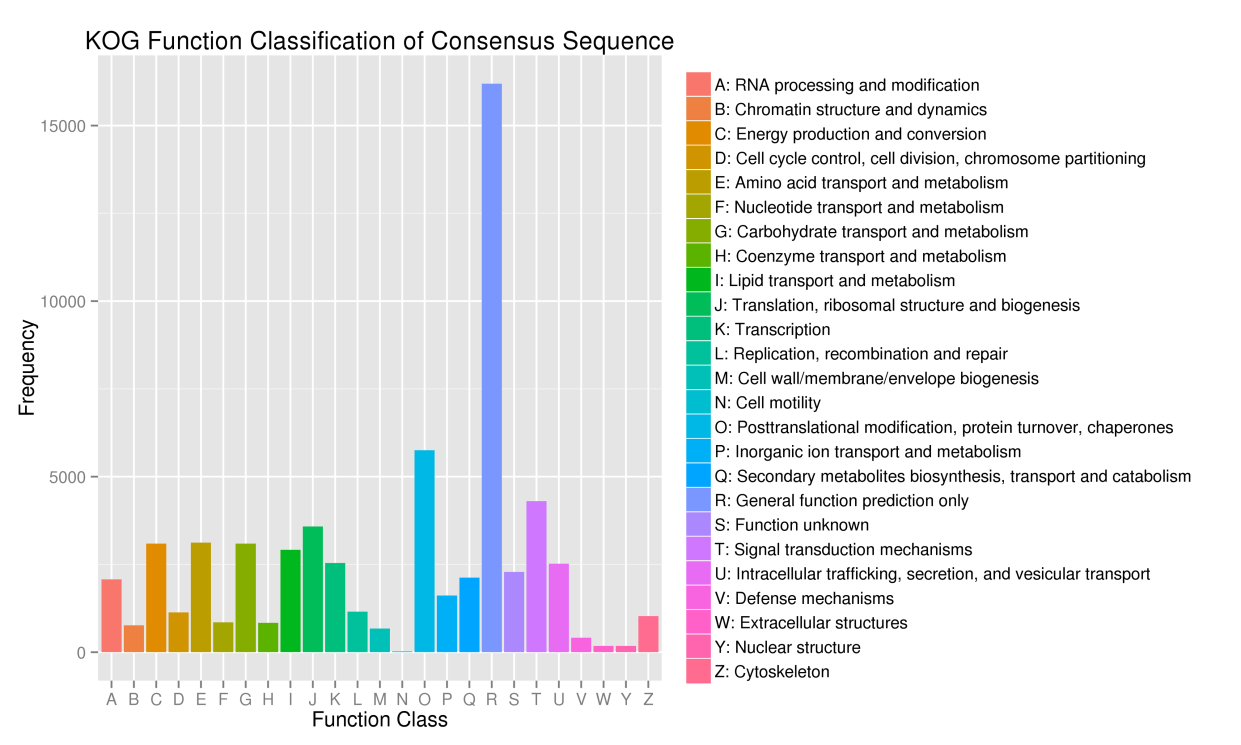


**Supplementary Figure 2**


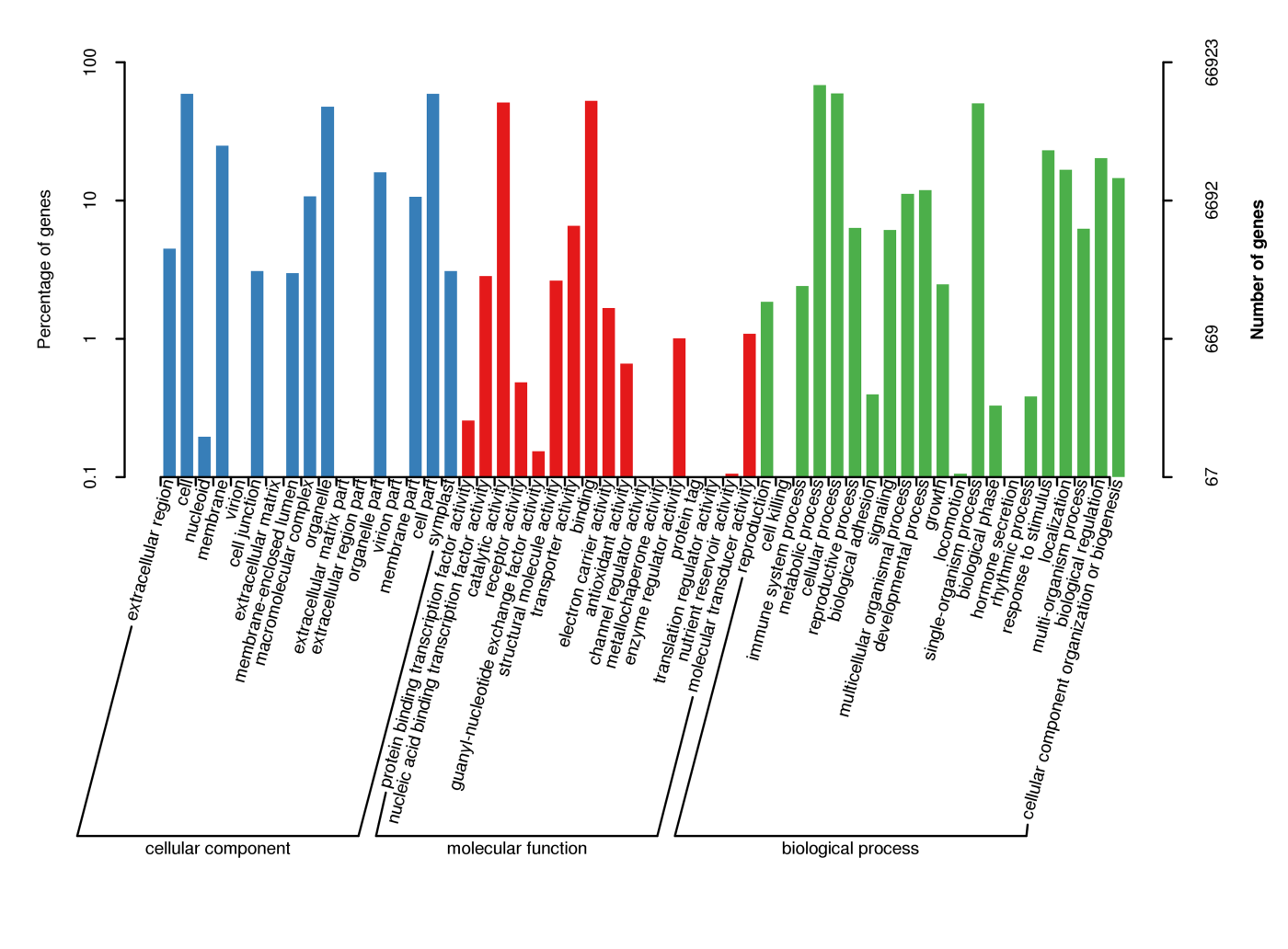


**Supplementary Figure 3**

**
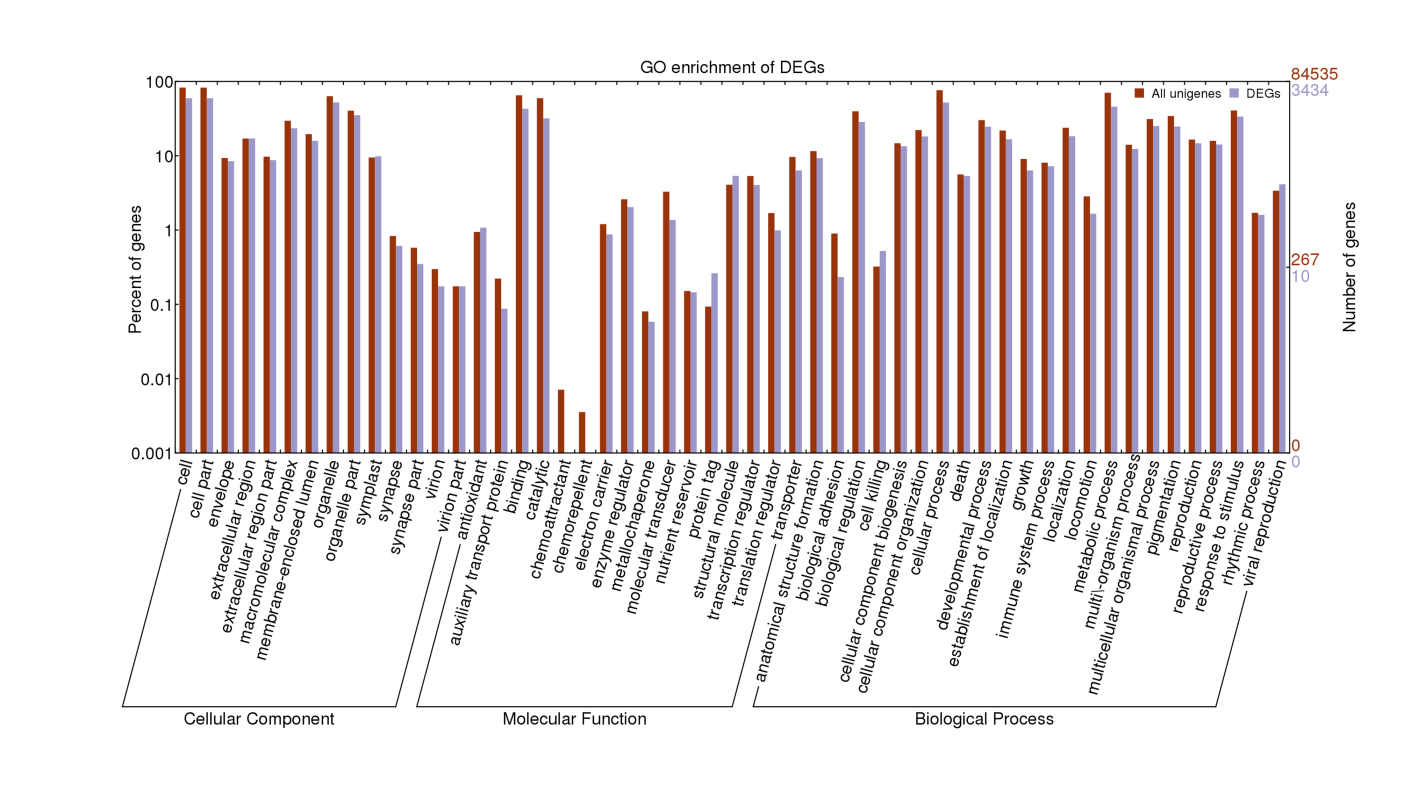
**

**Supplementary Figure 4**

**
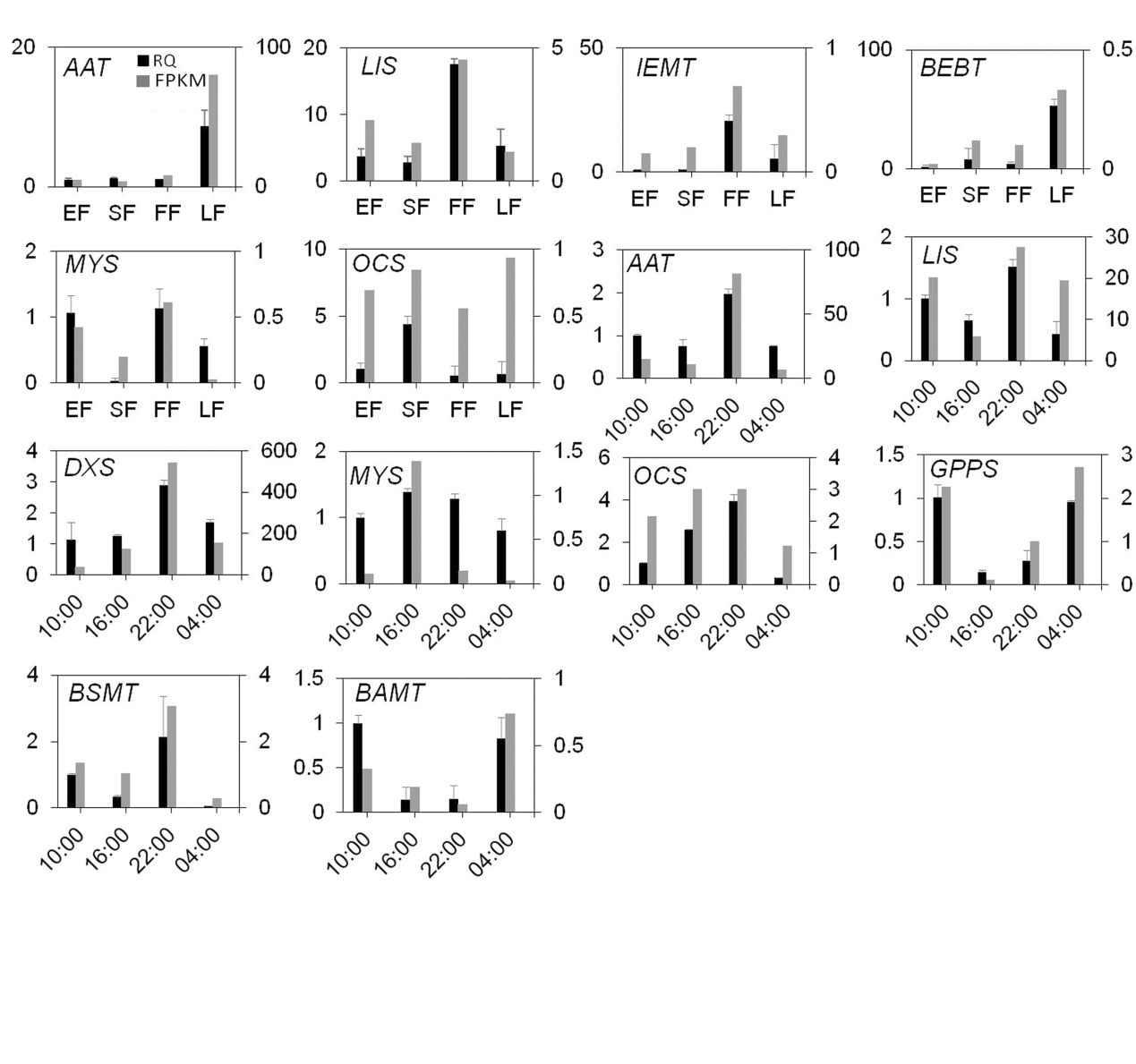
**

**Supplementary Figure 5**

**
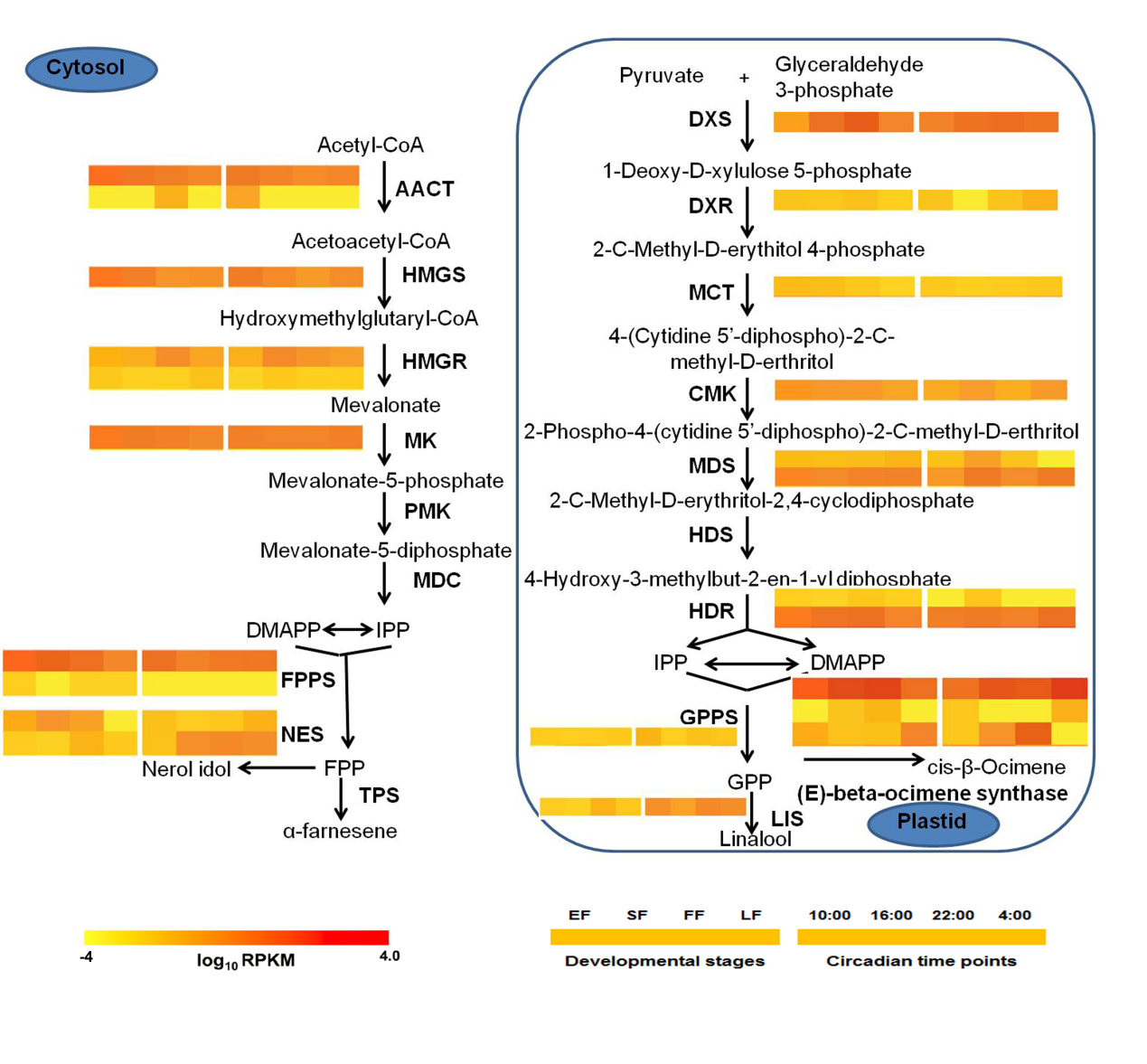
**

**Supplementary Figure 6**

**
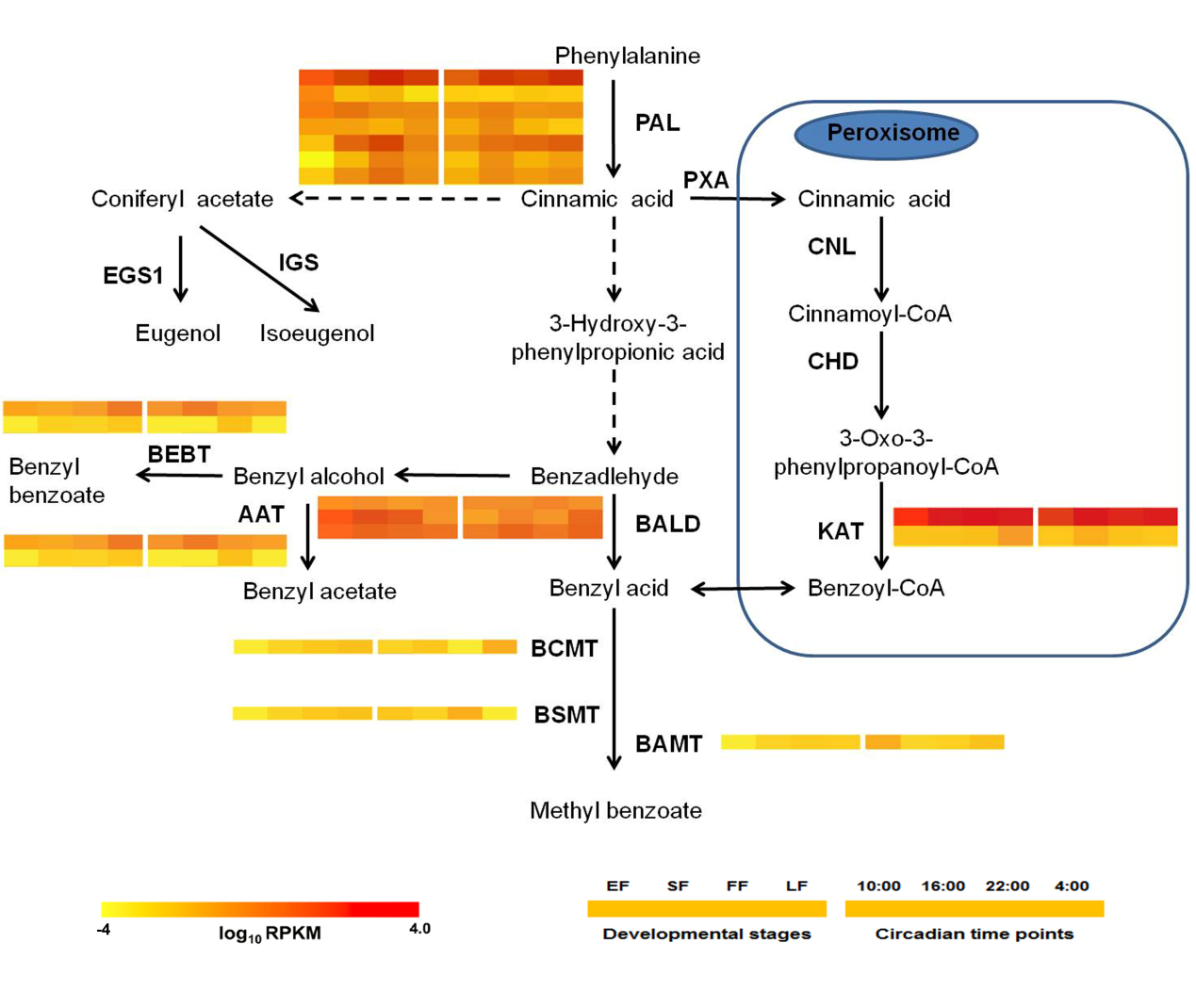
**

**Supplementary Figure 7**

**
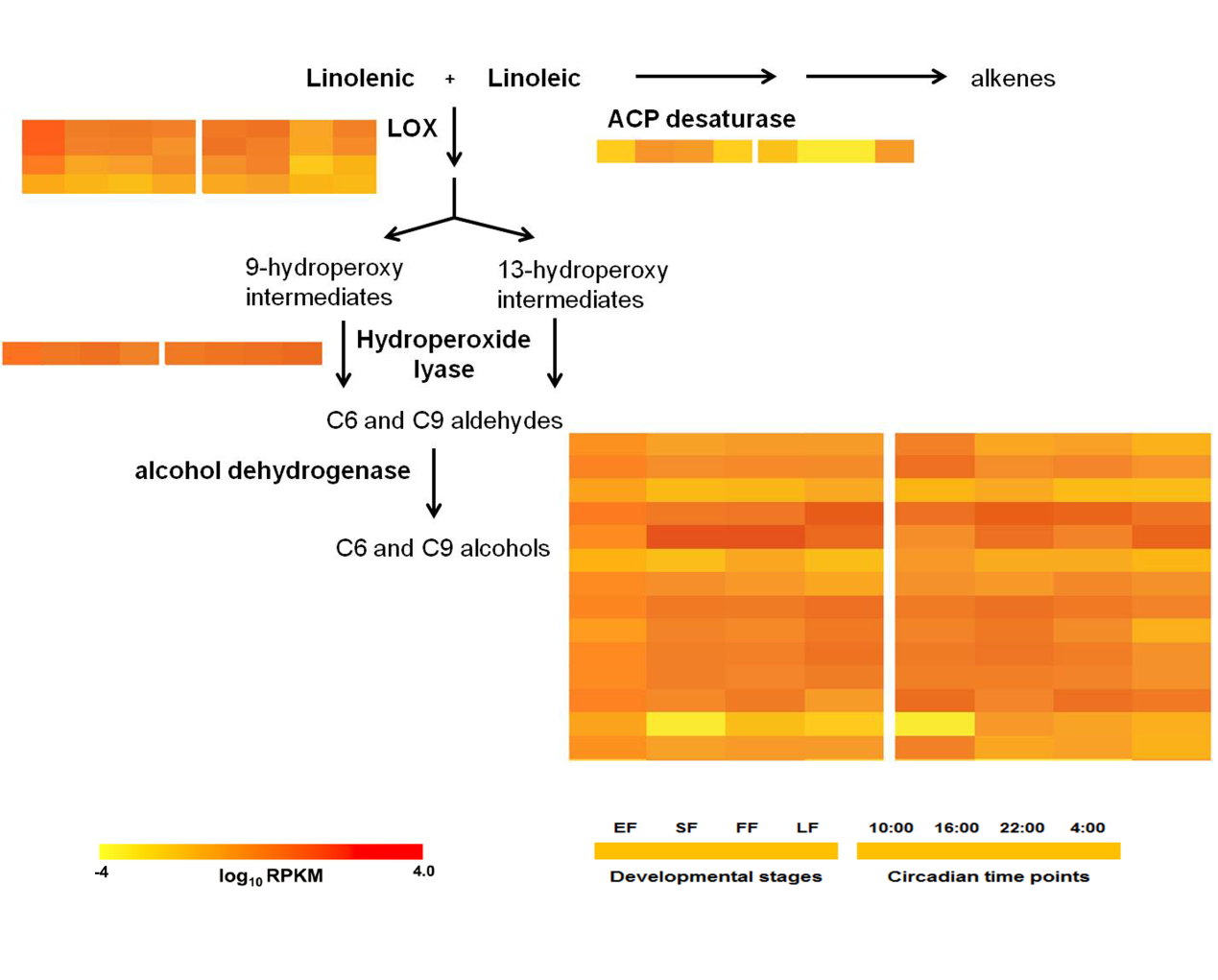
**

**Supplementary Figure 8**

**
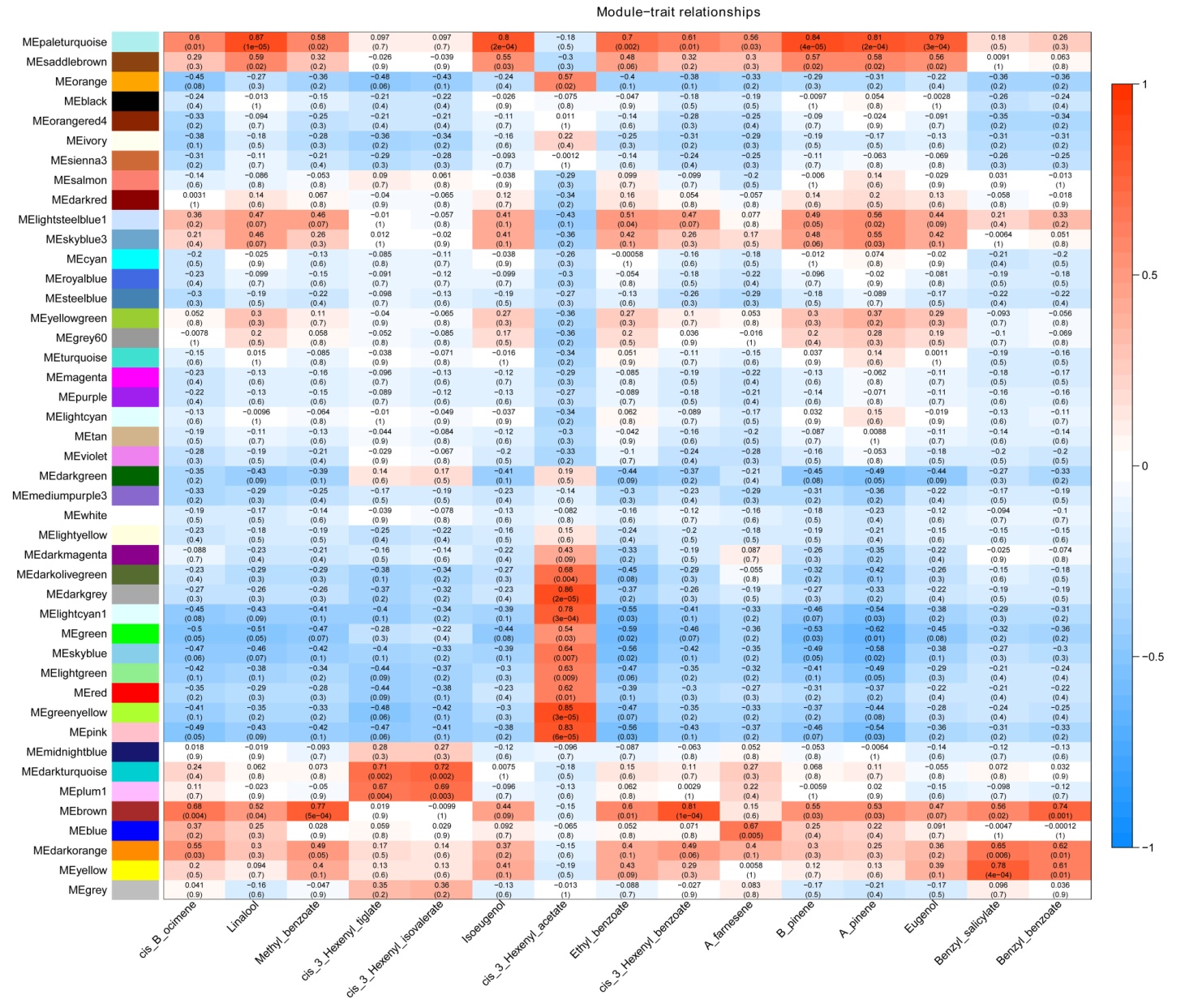
**

**Supplementary Figure 9**

**
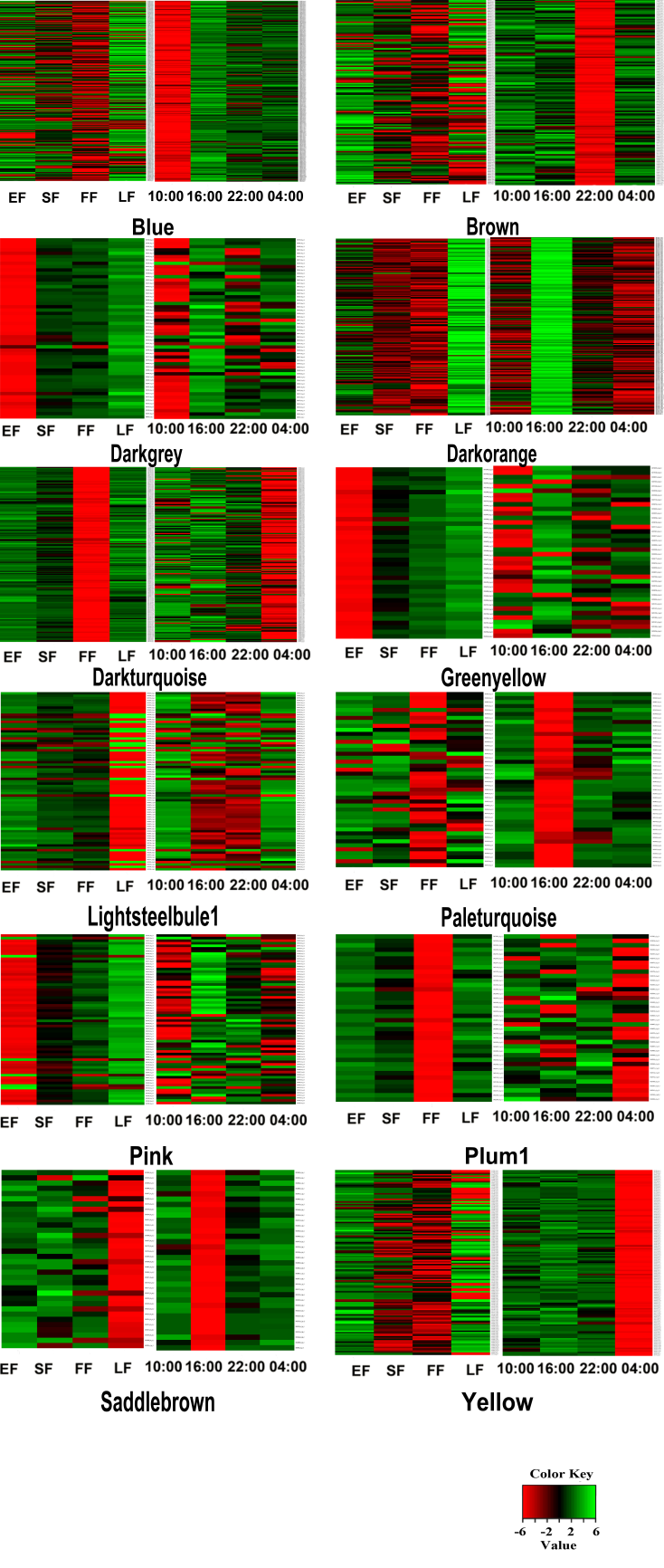
**

**Supplementary Dataset (uploaded as excel file)**

**Table S1 | Primers used in Q-PCR validation for RNA-seq data.**

**Table S2 | Floral volatile changes during flower development process in *Lilium* 'Siberia'.**

**Table S3 | Floral volatile emission changes across flower developmental stages and time of day in *Lilim* 'Siberia'.**

**Table S4 | KEGG pathways mapped by unigenes.**

**Table S5 | Genes encoding enzymes possibly involved in flower scent biosynthesis in *Lilium* 'Siberia'**

**Table S****6 | Annotation of genes in modules.**

**Table S7 | Annotation of DEGs in modules.**

**Table S8 | Annotation of TFs in modules.**
